# Transcriptional Response in the Digestive Gland of the King Scallop (*Pecten maximus*) After the Injection of Domoic Acid

**DOI:** 10.3390/toxins13050339

**Published:** 2021-05-07

**Authors:** Pablo Ventoso, Antonio J. Pazos, Juan Blanco, M. Luz Pérez-Parallé, Juan C. Triviño, José L. Sánchez

**Affiliations:** 1 Departamento de Bioquímica y Biología Molecular, Instituto de Acuicultura, University of Santiago de Compostela, 15782 Santiago de Compostela, Spain; pabloventoso24@hotmail.com (P.V.); luz.perez-paralle@usc.es (M.L.P.-P.); joseluis.sanchez@usc.es (J.L.S.); 2 Centro de Investigacións Mariñas, Xunta de Galicia, Pedras de Corón s/n Apdo. 13, 36620 Vilanova de Arousa, Spain; juan.carlos.blanco.perez@xunta.gal; 3 Sistemas Genómicos, Ronda G. Marconi 6, Paterna, 46980 Valencia, Spain; jc.trivino@sistemasgenomicos.com

**Keywords:** amnesic shellfish poisoning (ASP), bivalves, RNA-seq, transcriptome, differential expression, DEGs, injection

## Abstract

Some diatom species of the genus *Pseudo-nitzschia* produce the toxin domoic acid. The depuration rate of domoic acid in *Pecten maximus* is very low; for this reason, king scallops generally contain high levels of domoic acid in their tissues. A transcriptomic approach was used to identify the genes differentially expressed in the *P. maximus* digestive gland after the injection of domoic acid. The differential expression analysis found 535 differentially expressed genes (226 up-regulated and 309 down-regulated). Protein–protein interaction networks obtained with the up-regulated genes were enriched in gene ontology terms, such as vesicle-mediated transport, response to stress, signal transduction, immune system process, RNA metabolic process, and autophagy, while networks obtained with the down-regulated genes were enriched in gene ontology terms, such as response to stress, immune system process, ribosome biogenesis, signal transduction, and mRNA processing. Genes that code for cytochrome P450 enzymes, glutathione S-transferase theta-1, glutamine synthase, pyrroline-5-carboxylate reductase 2, and sodium- and chloride-dependent glycine transporter 1 were among the up-regulated genes. Therefore, a stress response at the level of gene expression, that could be caused by the domoic acid injection, was evidenced by the alteration of several biological, cellular, and molecular processes.

## 1. Introduction

Some diatom species of the genus *Pseudo-nitzschia* produce domoic acid, a toxin that can cause amnesic shellfish poisoning (ASP) in humans [1,2,3,4,5]. During harmful algae blooms the bivalves accumulate the toxins in their tissues and therefore they can act as vectors of ASP [1,2,3]. The accumulation of biotoxins in shellfish can cause harvesting closures and thus it has adverse economic impacts. In recent years there has been an increase in the number of toxigenic *Pseudo-nitzschia* blooms worldwide [2,6]. Domoic acid, an amino acid structurally similar to glutamate and kainic acid, is a glutamate receptor agonist that binds mainly to two subtypes of ionotropic receptors (α-amino-3-hydroxy-5-methylisoxazole-4-propionate, AMPA, and kainate receptors [4,7,8]) and exerts excitotoxic effects in the central nervous system of vertebrates [4,7,8].

The king scallop *Pecten maximus* is a valuable fisheries resource in Europe [9]. Unlike mussels and oysters [10,11,12,13], with high domoic acid depuration rates, in the king scallop (*Pecten maximus*) the depuration rate of domoic acid is very low [14,15]. Due to the blooms of domoic acid-producing species of the genus *Pseudo-nitzschia* and the low depuration rate of *P. maximus* [14,15], the domoic acid concentration in this scallop is usually above the regulatory limits (20 µg of domoic acid g^−1^) in many areas [16]. There are other scallop species (*Placopecten magellanicus* and *Argopecten purpuratus*) that do not show this slow domoic acid depuration [17,18].

Domoic acid in bivalves is mostly unmetabolized and is excreted unchanged [10]. In many bivalves, including the king scallop, the digestive gland is the main organ of accumulation of domoic acid [10,14,15,17,19,20]. In the digestive gland of *P. maximus*, domoic acid was found to be present in the cytosol in soluble form [21], therefore Mauriz and Blanco [21] suggested that the lack of an efficient membrane transporter could be the cause of the low depuration rate. In a recent work, Blanco et al. [16] showed that this toxin, in the digestive gland, is mainly accumulated in large cells (digestive cells) and the concentration was lower in small cells (secretory cells).

Several publications have described the physiological effects of domoic acid and domoic acid-producing organisms on marine bivalves: transient DNA damage in *Mytilus edulis* after de injection of domoic acid [22], mild respiratory alkalosis in *Mytilus californianus* after the exposure to domoic acid-producing *Pseudo-nitzschia* [23], transient respiratory acidosis, hypoxia, and increase in the activity and number of haemocytes in *Crassostrea gigas* following the exposure to domoic acid-producing *Peseudo-nitzschia* [23,24], negative impacts on survival and growth rate in juvenile *Pecten maximus* exposed to domoic acid-spiked feed [25], negative effects on growth and survival in *Pecten maximus* larvae exposed to dissolved domoic acid [26], impairment of immune functions and oxidative stress in *Argopecten irradians* exposed to dissolved domoic acid [27,28]. In two previous works, we studied the transcriptional effects of domoic acid-containing *Pseudo-nitzschia* in the digestive gland of the mussel *Mytilus galloprovincialis* [29] and the queen scallop *Aequipecten opercularis* [30] by means of RNA-seq technology. The results obtained showed the differential expression of genes involved in protection against oxidative stress, the metabolism of xenobiotics (detoxification), transmembrane transport, and immunological processes [29,30]. Oxidative stress is one of the principal effects caused by exposure to domoic acid in vertebrates [31,32,33,34], and also in invertebrates [35,36], including marine bivalves (*Argopecten irradians* [28] and *Aequipecten opecularis* [30]), and can play an important role in domoic acid induced toxicity. In the present work, a transcriptomic approach (RNA-seq) has been used to identify the genes differentially expressed in the digestive gland of the king scallop *P. maximus* after the injection of domoic acid, in order to uncover dysregulated biological and molecular processes and contribute to the knowledge of detoxification mechanisms in bivalves.

## 2. Results

### 2.1. Domoic Acid Content in the Tissues of P. maximus

**Figure 1:**
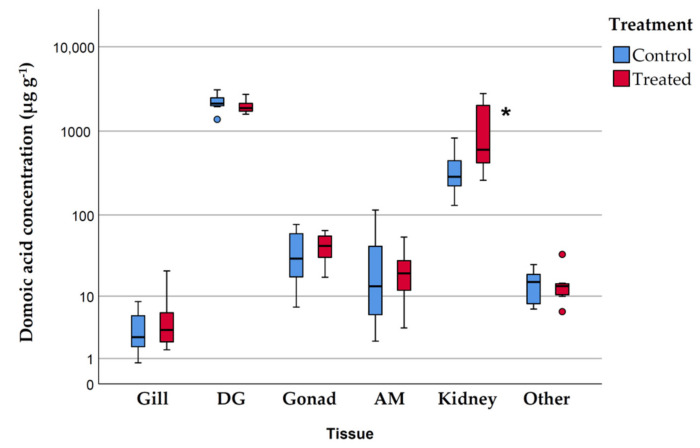
Box plots showing the domoic acid concentration (µg g^−1^ wet weight) in control and treated scallops (*Pecten maximus*). DG, digestive gland; AM adductor muscle; Other, remaining tissues. * significant difference from control (*t*-test, *p* < 0.05).

Table 1 and Figure 1 display the domoic acid concentration (µg g^−1^wet weight) and burden (µg) in different *P. maximus* tissues. Due to the low depuration rate of *P. maximus* [14,15], and the blooms of toxigenic *Pseudo-nitzschia*, the king scallops collected in Galicia (NW Spain) have high levels of domoic acid in their body tissues. This explains why domoic acid concentration in the digestive gland (DG) is high in control and treated scallops (2222.9 and 1987.0 µg g^−1^ wet weight, respectively). The domoic acid burden in the digestive gland of control and treated animals was 3690.2 µg and 3884.8 µg, respectively. The domoic acid content in the digestive gland accounted for 84% of the total domoic acid burden in the body tissues of *P. maximus*, despite the fact that the digestive gland only represented about 7% of the total weight of soft tissues.

The kidney (Table 1 and Figure 1) is the only organ that shows a clear increase in the concentration of domoic acid (and in the domoic acid burden) in treated animals (1226.34 µg g^−1^ wet weight) in relation to the control (370.70 µg g^−1^ wet weight). In absolute terms, the greatest increases in the domoic acid burden were found in the kidney followed by the digestive gland (Table 1). Significant differences between control and treated animals were found only in the concentration of domoic acid in the kidney (log10 transformed to meet the assumptions of normality and homoscedasticity; t-test, *p* = 0.041).

Although the experiment was not designed to see the effect of domoic acid on the behavior of scallops, we have observed that the scallops treated with domoic acid maintained a larger valve opening than those injected with seawater, but no mortality was recorded.

### 2.2. Sequencing and De Novo Assembly

The reads obtained by means of paired-end sequencing in the Illumina Hiseq 2000 platform were de novo assembled with Trinity and Oasis. Then, to reduce redundancy, the assembled transcripts were clustered (homology > 90%), thus 72,673 unigenes were obtained (Table 2). Mean contig length and N50 contig length were 1481 bp and 1883 bp respectively (Table 2). The raw data are accessible from the NCBI Sequence Read Archive (BioProject ID PRJNA704533, BioSample accessions: SAMN18043529 to SAMN18043540).

A BUSCO analysis of completeness of the de novo assembly identified 82.29% complete orthologs (45.6% single-copy and 36.69% duplicated), 3.88% fragmented orthologs and 13.83% missing orthologs (Table S1). The BUSCO results indicate that our de novo transcriptome assembly is of high quality, and although we only sequenced one tissue (digestive gland) the assembly has a completeness of 86.2%.

### 2.3. Differential Expression and Functional Annotation

A representation of the differentially expressed genes (DEGs) is displayed in a MA plot (Supplementary Figure S1) and a volcano plot (Supplementary Figure S2). The number of genes with absolute fold change > 1.5 and adjusted *p*-value < 0.05 (DEGs) was 535, of which 226 were up-regulated and 309 down-regulated (Supplementary Files S1 and S2). Table 3 and Table 4 display the top 20 significantly up-regulated genes and the top 20 significantly down-regulated genes, respectively.

Among the top up-regulated genes (Table 3, Supplementary File S1) were genes involved in the metabolism of xenobiotics (cytochromes P450, glutathione S-transferase) a gene (*SLC6A9*) coding for a sodium- and chloride-dependent glycine transporter (a solute carrier of the *SLC6* family [37]) and two genes coding for enzymes involved in the metabolism of glutamate and proline (glutamine synthetase and pyrroline-5-carboxylate reductase 2). Pinin, serine protease inhibitor Cvsi-1, innexin UNC-7, serine/threonine-protein kinase PINK1 and apoptosis-stimulating of p53 protein 1 were among the top down-regulated genes (Table 4). Many of the top down-regulated genes code for uncharacterized proteins (Table 4).

It is worth pointing out that we have found 19 transcripts that code for putative glutamate receptors in the digestive gland of *P. maximus*, 10 of them for ionotropic receptors and 9 for metabotropic receptors (Table 5 and Supplementary File S3), but none of these genes were differentially expressed.

The functional annotation results are shown in Table 6 and in Supplementary Files S4 and S5.

### 2.4. Protein Network Analysis

**Figure 2:**
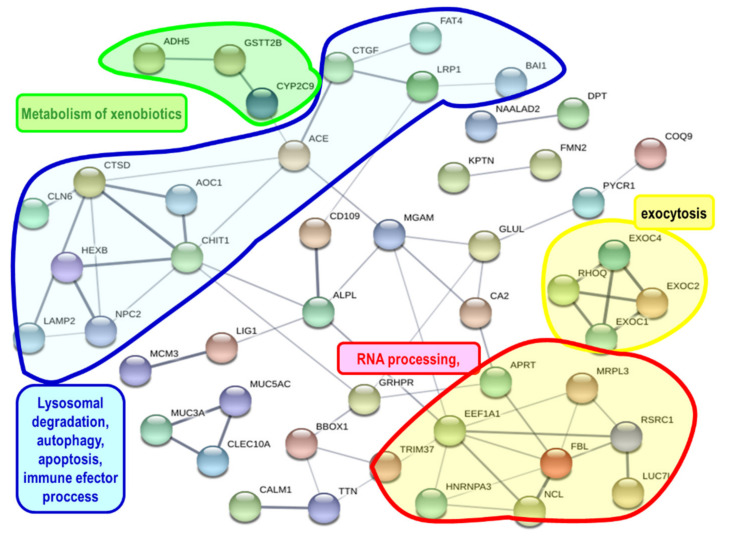
Network showing interactions of proteins coded by genes up-regulated in the present study. The network was constructed using the String 10.5 algorithm. Some connected protein nodes are highlighted showing some of the processes in which these proteins participate. Proteins were named according to the human protein name. A full list of protein names is available in Supplementary File S6.

**Figure 3:**
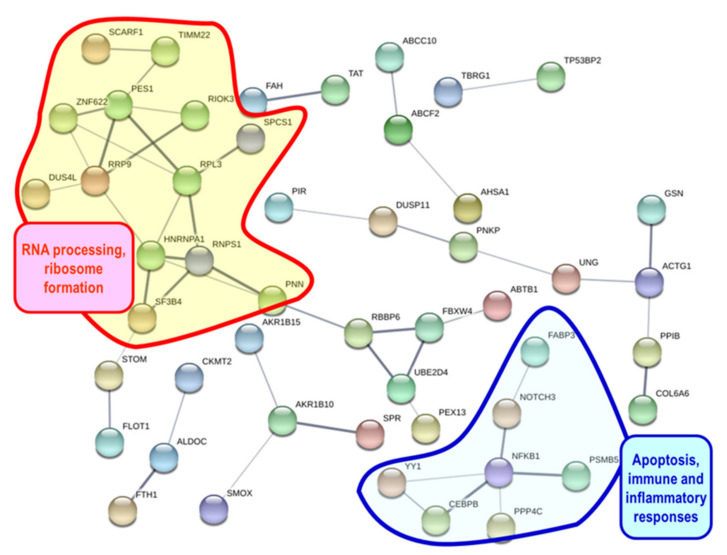
Network showing interactions of proteins coded by genes down-regulated in the present study. The network was constructed using the String 10.5 algorithm. Some connected protein nodes are highlighted showing some of the processes in which these proteins participate. Proteins were named according to the human protein name. A full list of protein names is available in Supplementary File S7.

Differentially expressed protein-coding genes can be grouped by means of the protein-protein interactions [38]. A Blastx search of the sequences of the 226 up-regulated and the 309 down-regulated genes found 88 and 93 human homologs in the STRING database, respectively. The networks obtained with up-regulated (Figure 2 and Supplementary File S6) and down-regulated (Figure 3 and Supplementary File S7) genes were enriched in interactions (*p*-values 5.31 × 10^−6^ and 0.0152, respectively). Up-regulated genes (Figure 2 and Supplementary File S6) were involved in biological processes, such as the metabolism of xenobiotics, stress response, immune response, lysosomal degradation, autophagy, apoptosis, RNA processing, and exocytosis. On the other hand, down-regulated genes (Figure 3 and Supplementary File S7) were involved in biological processes such as RNA processing, ribosome formation, apoptosis, immune and inflammatory responses.

The up-regulated genes that code for proteins that showed interactions in the protein network analysis were enriched in gene ontology (GO) terms (Supplementary File S8) such as vesicle-mediated transport, response to stress, signal transduction, immune system process, RNA metabolic process, autophagy, lysosome and oxidoreductase activity. The down-regulated genes that code for proteins that showed interactions in STRING analysis were enriched in GO terms (Supplementary File S8) such as response to stress, immune system process, ribosome biogenesis, signal transduction, mRNA processing, and oxidoreductase activity.

### 2.5. Real Time RT-qPCR

Four candidate reference genes (*GAPDH*, *EF1A*, *COX1* and *NDUFA7*, Table 7) were selected based on a previous work on *P. maximus* [39]. Table 8 shows the results obtained with the three algorithms (geNorm, NormFinder and BestKeeper) employed to test the stable expression of these genes. *GAPDH*, and *COX1* were the best ranked genes and therefore selected as reference genes.

**Figure 4:**
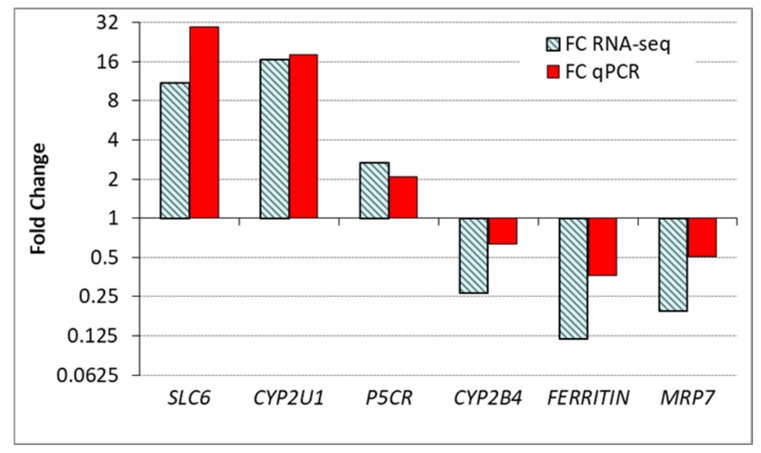
Gene expression (fold change in relation to control) in the digestive gland of *P. maximus* as determined by RT-qPCR analyses and RNA-seq. FC qPCR = geometric mean of the normalized gene expression in treated samples/geometric mean of the normalized gene expression in control samples.

The comparison of gene expression results of the six target genes (*MRP7, CYP2B4, P5CR, SLC6A9, FERRITIN, CYP2U1*, Table 7) obtained with RT-qPCR and RNA-seq is displayed in Figure 4. The log2 FC values achieved by the two methods were very similar and showed good correlation (*r* = 0.969; *r*^2^ = 0.939).

## 3. Discussion

Several authors have shown [14,15,16] that the digestive gland in the king scallop *P. maximus* accumulates most of the domoic acid. This agrees with the results obtained in the present work that found that domoic acid content in the digestive gland accounts for 84% of the total domoic acid burden in the body tissues of *P. maximus* (Table 1). The total amount of domoic acid injected was 3000 µg per animal, but only a mean difference of 339 µg between treated and control animals was found. These results suggest that part of the administered domoic acid was depurated. After the injection into the adductor muscle, the domoic acid enters into the circulatory system, through the hemolymph sinuses [40]. In scallops, urine formation takes place through hemolymph filtration into the pericardial cavity, which in turn is connected to the kidneys through the reno-pericardial ducts [40]. Therefore, part of the domoic acid is probably filtered from the hemolymph into the kidneys and only a part of the injected domoic acid was distributed to the digestive gland. In kidneys, we have found a significant difference in domoic acid concentration between control and treated animals (Figure 1). It is important to emphasize that in the natural environment the scallops obtain the toxin from the food-chain through the digestive system.

We found 535 DEGs (226 up-regulated and 309 down-regulated, Supplementary File S1) in the digestive gland of *P. maximus* after the injection of domoic acid, therefore the toxin might have an effect on the gene expression in the digestive gland. The analysis of gene expression found the alteration of some processes at the biological, cellular, and molecular level (Figure 2 and Figure 3, Supplementary File S8), that could be due to the effects of domoic acid. Thus, genes involved in xenobiotic metabolism, immune response, response to stress, signal transduction, apoptosis, RNA processing, ribosome biogenesis, lysosomal degradation, autophagy, and regulated exocytosis were differentially expressed. Scallops injected with domoic acid showed a behavior different (maintained a clearly larger valve opening) to the ones to which only seawater was injected. This reveals that domoic acid probably had an effect at the level of the central nervous system. There are three ways, not mutually exclusive, that can explain how domoic acid exerts its effects on the digestive gland of *P. maximus*: (a) the interaction of domoic acid with different biomolecules (mainly proteins) after the entry of domoic acid into the cells of the digestive gland; (b) the binding of domoic acid to glutamate receptors present on the plasma membrane of the cells of the digestive gland (we found that there is expression of mRNA that codes for glutamate receptors, both ionotropic and metabotropic, in the digestive gland of *P. maximus*, Table 5 and Supplementary File S3); and (c) the binding of domoic acid to glutamate receptors present in the central nervous system (cerebral, pedal, and parietovisceral ganglia) of the scallop (the actions triggered in these ganglia could be transmitted to the digestive gland through the nervous and neuroendocrine systems). Dizer et al. [22] also found an effect (increased DNA damage) of the intramuscular injection of domoic acid on digestive gland cells of a bivalve, *Mytilus edulis*.

In vertebrates, domoic acid is a potent neurotoxin [4,8,41,42,43], and the response to domoic acid includes genes involved in transcription (transcription factors), signal transduction, ion transport, generalized response to stress, mitochondrial function, inflammatory response, response to DNA damage, apoptosis, neurological function and neuroprotection [31,41,44,45]. Although there are fewer studies on the effects of domoic acid on invertebrates than vertebrates, this toxin also exerts effects on marine bivalves at the physiological and gene expression levels [22,23,24,25,26,28,29,30]. In two previous studies [29,30] we showed that exposure to domoic acid containing *Pseudo-nitzschia* alters the transcriptomic profile of the digestive gland of the mussel *Mytilus galloprovincialis* and of the queen scallop *Aequipecten opercularis*. The results obtained by Ventoso et al. [30] suggest that exposure to domoic acid-producing organisms causes oxidative stress and mitochondrial dysfunction in *A. opercularis*, thus the transcriptional response of the queen scallop is involved in the protection against oxidative stress. This agrees with the results obtained by Song et al. [28] that showed that domoic acid induces oxidative stress and impairs defence mechanisms in bay scallops (*Argopecten irradians*). The contribution of oxidative stress to the effects and toxicity of domoic acid has been highlighted by several authors [6,28,31,32,33,35,36].

A consequence of oxidative stress, if the protective anti-oxidant mechanisms cannot limit the damage, is cellular dysfunction and apoptosis [46], and domoic acid can induce apoptosis [32,47,48,49]. Cathepsin D, a lysosomal aspartic acid protease that initiates caspase-8-dependent apoptosis [50], was up-regulated in *P. maximus* (Figure 2 and Supplementary File S1), and also in *A. opercularis* [30] and *M. galloprovincialis* [29] after exposure to domoic acid containing *Pseudo-nitzschia*. Several genes coding for proteins putatively involved in apoptosis were differentially expressed in *P. maximus* (CTSD, AOC1, LRP1, BAI1, NFKB1, NOTCH3, PPP4C, RBBP6, Figure 2 and Figure 3).

One of the effects of domoic acid in *P. maximus* was the down-regulation of genes involved in RNA processing, ion transport, immune response, metabolic process and signal transduction (Supplementary File S8); this agrees with the results of Lefebvre et al. [41] with zebrafish, after low-level domoic acid exposures, that found the down-regulation of genes involved in those same biological processes.

Genes coding for several phase I (cytochromes P450) and phase II (glutathione S-transferases and sulfotransferases) drug metabolizing enzymes were up-regulated in *P. maximus* (Table 3, Figure 2 and Supplementary File S1), these types of genes were also up-regulated in *A. opercularis* [30] and *M. galloprovincialis* [29] following exposure to domoic acid-producing *Pseudo-nitzschia*.

Several authors have shown that glutamate receptors are expressed not only in the central nervous system but also in other types of tissues or organs (intestine, liver, kidney, stomach, etc.) [51,52,53]. Therefore, glutamate and glutamate receptor agonists could participate in the regulation of several physiological processes in peripheral organs [51,52,53]. We have found 19 genes that code for possible glutamate receptors in the digestive gland of *P. maximus*, 10 of them for ionotropic receptors and 9 for metabotropic receptors (Table 5 and Supplementary File S3). Some of the effects of domoic acid on the cells of the digestive gland may be mediated by these receptors. None of the genes coding for these receptors were differentially expressed in *P. maximus* (Table 5 and Supplementary File S3). In *A. opercularis*, some genes coding for glutamate ionotropic receptors were down-regulated [30] in the digestive gland of animals exposed to domoic acid-containing *Pseudo-nitzschia*. This may be due to a compensatory response to elevated glutamatergic activity, thus Hiolski et al. [31] found this type of compensatory response in zebrafish after domoic acid exposure.

Glycine, in addition to acting as an inhibitory neurotransmitter, is also a co-agonist at N-methyl-D-aspartate (NMDA) glutamate receptors [54]. In the central nervous system of vertebrates, the glycine transporter 1 (sodium- and chloride-dependent glycine transporter 1) regulates the binding of glycine to NMDA receptors [54], because the action of glycine is terminated through the reuptake mediated by sodium- and chloride-dependent glycine transporters [55]. The up-regulation of the *SLC6A9* gene (coding for sodium and chloride-dependent glycine transporter 1) could prevent or reduce NMDA receptor activation. The *SLC6A9* gene was among the top up-regulated genes in *P. maximus* (Table 3). There was another gene of this family (*SLC6*) that was downregulated in *P.maximus* (Supplementary File S1). Although both genes code for putative sodium- and chloride-dependent glycine transporters, they share only 52% sequence identity at the amino acid level. Genes of this family (*SLC6*) were up-regulated in *M. galloprovincialis* [29] and down-regulated in *A. opercularis* [30] after exposure to domoic acid-producing *Pseudo-nitzschia*. A gene of the *SLC6* family was up-regulated in *Pseudo-nitzschia multiseries* under toxin-producing conditions [56], and this gene was also up-regulated in a domoic acid-producing *Pseudo-nitzschia* species in relation to two *Pseudo-nitzschia* species that do not produce domoic acid [57]. The *SLC6* family is expanded in the genome of the scallops *Chlamys farreri and Patinopecten yessoensis* [58,59], in relation to other bivalves. In the *A. opercularis* [30] and in *P. maximus* digestive gland transcriptome, the number of transcripts belonging to this family is also very high (we found 58 in *P. maximus*).

One of the up-regulated genes in *P. maximus*, glutamine synthetase (Supplementary File S1), might play a neuroprotective role against glutamate neurotoxicity in neural tissues [60,61], because it catalyzes the transformation of glutamate to glutamine. Glutamate and glutamate receptor agonists increased glutamine synthetase expression and glutamine synthetase activity in cultured astrocytes [62,63]. Glutamine synthetase also participates in the production of GABA (gamma-aminobutyric acid), an inhibitory neurotransmitter. GABA has been shown to be able to prevent, at least partially, the effects of domoic acid in rat glial cells [64]. Therefore, the overexpression of this gene could have a protective effect against domoic acid. Another gene involved in the metabolism of amino acids (glutamate and proline) is up-regulated in *P. maximus*. This gene codes for the enzyme pyrroline-5-carboxylate reductase 2 that catalyzes the conversion of pyrroline-5-carboxylate to proline, and proline has a protective effect against oxidative stress [65]. Kenny et al. [66] suggested that mutations in the sodium channel gene, *Neuron Navigator 1* (*Nav1*), could protect against the effects of domoic acid in *P. maximus*. We have not found transcripts of the *Nav1* gene in the digestive gland of *P. maximus*, therefore the sodium channel is likely expressed in nervous tissue but not in the digestive gland.

The immune system of marine bivalves is sensitive to toxins and harmful algae [67,68]. Several harmful algae can provoke a stimulation of immune functions, while others cause inhibition [68]. Chi et al. [27] found that exposure to domoic acid impaired immune functions in the bay scallop *A. irradians*. In *P. maximus*, the immune system process was one of the enriched terms in the proteins coded by DEGs that showed interaction in STRING (Figure 2 and Figure 3, Supplementary Files S6–S8). C-type lectins are calcium-dependent carbohydrate-binding proteins and participate in innate immunity in bivalves [67,69]. In bivalve mollusks there is a high number of genes coding for C-type lectins [69,70]. Three transcripts coding for putative C-type lectins were up-regulated in the *P. maximus* digestive gland (Supplementary File S1). This agrees with the results obtained with *A. opercularis* [30] and *M. galloprovincialis* [29] after exposure to domoic acid-producing *Pseudo-nitzschia*, where most of the genes coding for C-type lectins were up-regulated.

Heat shock proteins (HSP) can be induced by several types of stress (high temperature, hypoxia, toxins, or pathogens) and they are involved in protein folding [71]. There is an expansion of *Hsp70* (heat shock protein 70 kDa) genes from the *Hspa12* subfamily in *Mizuhopecten yessoensis* [71], and a gene of this subfamily (*heat shock 70 kDa protein 12A-like*) was up-regulated in *P. maximus* (Supplementary File S1). A up-regulation of *HSP* genes after exposure to harmful algae toxins (including domoic acid) has been found in several bivalves [27,28,71,72,73,74,75], although in *A. opercularis*, after exposure to domoic acid-producing *Pseudo-nitzschia*, HSP genes were both up- and down-regulated [30].

The digestive gland of *Pecten maximus* contains principally two types of cells, secretory cells and digestive cells [16,40,76,77]. Vesicle-mediated transport and regulated exocytosis were enriched biological processes identified in protein networks obtained with the up-regulated genes (Figure 2 and Supplementary File S8), therefore the activity of secretory cells in the digestive gland may be stimulated by domoic acid. These cells may be involved in the secretion of digestive enzymes [40,76,77].

Collagens are structural components of the extracellular matrix [78]. Components of collagen and proteins involved in cytoskeleton dynamics were among the proteins that appeared in the network obtained with the down-regulated genes in *A. opercularis* after exposure to domoic acid-producing *Pseudo-nitzschia* [30]. In the present work, several collagen genes were down-regulated (File S1) and cytoskeleton (Supplementary File S8) is one of the enriched cellular components in the down-regulated genes coding for proteins that showed interactions in the protein network analysis.

## 4. Conclusions

The domoic acid injected might have an effect on the gene expression in the digestive gland as reflected in the 535 DEGs found (226 up-regulated and 309 down-regulated). Some genes that code for putative glutamate receptors were expressed in the digestive gland of *P. maximus*, therefore part of the effects of domoic acid may be mediated by these receptors. A stress response at the level of gene expression, that could be caused by the domoic acid injection, was evidenced by the alteration of several biological, cellular, and molecular processes. Thus, protein networks obtained with the up-regulated genes were enriched in gene ontology (GO) terms, such as vesicle-mediated transport, response to stress, signal transduction, immune system process, RNA metabolic process, autophagy, lysosome, and oxidoreductase activity. On the other hand, networks obtained with the down-regulated genes were enriched in terms, such as response to stress, immune system process, ribosome biogenesis, signal transduction, mRNA processing, and oxidoreductase activity. In future research, it would be interesting investigate the domoic acid effects on gene expression in the kidneys and in the central nervous system (cerebral, pedal, and parietovisceral ganglia) of *P. maximus*.

## 5. Materials and Methods

The methods employed were similar as those previously described [29,30] except for minor modifications.

### 5.1. Animals

King scallops were obtained from the Ría de Arousa (Galicia, NW Spain) and maintained for a week in a 500 L tank, with a continuous unfiltered seawater flow (approximate) of 1200 L·h^−1^. No domoic acid was detected in the routine monitoring of mollusks from the area and no toxic *Pseudo-nitzschia* cells were present in the area neither during the experiment nor during the previous month. All scallops, notwithstanding, contained domoic acid. It was impossible to obtain individuals free of toxin from the study area (and even from other European Countries) because the very low depuration rate of this species [14] makes easy their re-contamination. In the data obtained by the monitoring system run by Intecmar during the last 25 years in Galicia, it has been observed that the prevalence of domoic acid in the king scallop was 100%, and the same happened in other areas, such as Scotland [79]. The experimental approach was conditioned by this limitation, so we try to induce a response in the scallops by increasing the domoic acid levels that, in natural conditions, only undergo the progressive and slow changes derived from the depuration process. Fourteen scallops, with average height 10.98 ± 0.16 cm and 24.9 ± 1.3 g of soft tissues weight, were randomized into two groups: seven scallops as a control group and the other seven as a treated (with toxin injections) group. The scallops in the treated group were subjected to repeated injections of domoic acid (ABCAM) dissolved in filtered seawater and those in the control group, to equivalent injections of filtered seawater. One injection was made into the adductor muscle of each scallop every other day, for a period of 12 days. A volume of 62.5 µL of filtered seawater with a concentration of 8 µg domoic acid µL^−1^ was used for each injection (500 µg domoic acid in each injection). The total amount of domoic acid injected was 3000 µg per animal. The same volume of filtered seawater was injected into the scallops in the control group. When the scallops spontaneously opened the valves, a silicone stopper was placed between them to maintain them opened, and the injection was carried out.

After the injection, each scallop (treatment and control) was placed into a 5 L beaker, with 4 L of seawater water, with aeration, and maintained there for 24 h. After that period, the scallops were transferred to a 500 L tank with running seawater for one day. Following that, the injection process was repeated. Twenty-four hours after the last injection the scallops were dissected into digestive gland, gill, kidney, gonad, adductor muscle and remaining tissues. The dissected tissues were used for the determination of domoic acid content using liquid chromatography-tandem mass spectrometry (LC–MS/MS). A portion of the digestive gland was treated with RNAlater (ref. AM7021, Ambion, Life Technologies) and stored at −80 °C until the RNA extraction.

### 5.2. Determination of the Domoic Acid Content

Methanol for HPLC and formic acid were purchased from Labscan (Bangkok, Thailand) and Sigma Aldrich (St. Louis, MO, USA), respectively. Ultrapure water was obtained using a Milli-Q Gradient system, coupled to an Elix Advantage 10, both from Millipore (Merck Millipore, Darmstadt, Germany).

To extract the toxin, each digestive gland was placed in aqueous methanol (50%) in a proportion of 1:2 *w:v* and homogenized with an Ultraturax T25 (IKA, Staufen, Germany). The extract was clarified using centrifugation at 18,000× *g* at 4 °C for 10 min, retaining the supernatant that was immediately analyzed.

Domoic acid in the obtained extracts was analyzed using LC–MS/MS. The chromatographic separation was carried out using a Thermo Accela chromatographic system (Thermo Fisher Scientific, Waltham, MA, USA), with a high-pressure pump and autosampler. The stationary phase was a solid core Kinetex C18, 50 × 2.1 mm 2.6 µm column (Phenomenex, Torrance, CA, USA). An elution gradient, with a flow of 280 µL min^−1^, was used with mobile phase A (formic acid 0.2%) and B (50% MeOH with formic acid 0.2%). The gradient started at 100% A, maintained this condition for one minute, linearly changed until reaching 55% B in minute 5, held for 2 min, and reverted to the initial conditions to equilibrate before the next injection. Five microliters of extract, previously filtered through a PES 0.2 µm syringe filter (MFS), were injected.

After the chromatographic separation, domoic acid was detected and quantified by means of a Thermo TSQ Quantum Access MAX triple quadrupole mass spectrometer (Thermo Fisher Scientific, Waltham, MA, USA), equipped with a HESI-II electrospray interface, using positive polarization and SRM mode. The transition 312.18 > 266.18 *m/z* was used to quantify the response and 312.18 > 248.18 for confirmation. The spectrometer was operated under the following conditions: spray voltage 3400 V, capillary temperature 270 °C, HESI-II temperature 110 °C, sheet gas (Nitrogen) 20 (nominal pressure), auxiliary gas (Nitrogen) 10 (nominal pressure), collision energy of 15 V and collision gas (Argon) pressure of 1.5 mTorr.

Concentrations of domoic acid were obtained by comparing the response of the quantification transition in the sample extracts with that of a reference solution obtained from NRC Canada. The quantification limit of the method for tissue analysis is less than 20 ng/mL of extract.

### 5.3. RNA Extraction

Twelve scallops (six obtained from the control group and six from the treated group) were subjected to RNA-seq analysis. A NucleoSpin RNA kit (ref. 740955, Macherey-Nagel, Düren, Germany) was used for digestive gland total RNA isolation. Then an RNA precipitation step with 0.5 volumes of Li CL 7.5 M was performed and the RNA pellet was dissolved in 50 µL of RNA storage solution (ref. AM7000, Ambion, Life Technologies, Carlsbad, CA, USA). Total RNA was treated with DNA-free (ref. AM1907M, Ambion, Life Technologies, Carlsbad, CA, USA) to remove DNA contamination. The integrity and quality of the RNA samples were measured using agarose gel electrophoresis, an Agilent 2100 Bioanalyzer (Agilent Technologies, Santa Clara, CA, USA) and a Nanodrop ND-1000 spectrophotometer (NanoDrop Technologies, Wilmington, DE, USA). The quantity of the total RNA was determined using Qubit 2.0 (Invitrogen, Carlsbad, CA, USA).

### 5.4. Library Preparation and Sequencing

Twelve cDNA libraries were generated. The poly(A)+ mRNA fraction was isolated from total RNA and cDNA libraries were obtained following Illumina’s recommendations. Briefly, poly(A)+ RNA was isolated on poly-T oligo-attached magnetic beads and chemically fragmented prior to reverse transcription and cDNA generation. The cDNA fragments then went through an end repair process, the addition of a single ‘A’ base to the 3′ end and afterwards the ligation of the adapters. Finally, the products were purified and enriched with PCR to create the indexed final double stranded cDNA library. The quality of the libraries was analyzed using a Bioanalyzer 2100 (Agilent Technologies, Santa Clara, CA, USA) high sensitivity assay; the quantity of the libraries was determined by real-time PCR in LightCycler 480 (Roche Diagnostics, Mannheim, Germany). Prior to cluster generation in cbot (Illumina), an equimolar pooling of the libraries was performed. The pool of the cDNA libraries was sequenced by paired-end sequencing (100 × 2 bp) on an Illumina HiSeq 2000 sequencer (Illumina, San Diego, CA, USA).

### 5.5. De Novo Assembly

Quality control checks of raw sequencing data were performed with FastQC. The technical adapters were eliminated using Trimgalore software version 0.3.3 (Trim Galore. Available Online: http://www.bioinformatics.babraham.ac.uk/projects/trim_galore/ (accessed on 10 March 2021)). Additionally, the reads with a mean Phred score > 30 were selected. Subsequently, the twelve samples were combined, and the complexity of the reads was reduced by removing duplicates. Then, a de novo assembly was performed using the programs Oasis, version 0.2.09 [80] and Trinity, version 2.1.1 [81]. The assembled transcripts were clustered (>90% homology) to reduce redundancy using cd-hit software version 4.6. For each sequence, the potential ORFs were detected using Transdecoder software, version 2.0, with standard parameters. The completeness of the de novo assembly was evaluated with BUSCO [82] in OmicsBox software (BioBam Bioinformatics—2019, Valencia, Spain. OmicsBox—Version 1.4.11.) [83,84], using the Metazoa database (metazoa_odb10) [85], with a total of 954 orthologs (Blast E-value < 10^−3^).

Each sample was then mapped with Bowtie2, version 2.2.6 [86] against the reference transcriptome obtained in the previous step. The good quality reads (Mapping Quality ≥ 20) were selected to increase the resolution of the count expression. Finally, the expression inference was evaluated by means of the counts of properly paired reads in each transcript.

### 5.6. Differential Expression

The transcriptome expression for each sample was normalized by library size, following the DESeq2 protocols. Differential gene expression analysis of treated versus control samples was performed with DESeq2 algorithm version 1.8.2 (DESeq2. Available online: http://www.bioconductor.org/packages/devel/bioc/html/DESeq2.html (accessed on 10 March 2021)). The genes with a fold change of less than −1.5 or greater than 1.5, and a *p*-value adjusted using the Benjamini and Hochberg [87] method for controlling false discovery rate (FDR) < 0.05 were considered differentially expressed.

### 5.7. Functional Annotation

Genes were annotated with OmicsBox software (BioBam Bioinformatics—2019. OmicsBox—Version 1.4.11.) [83,84], using local Blastx 2.10.0+ [88], (E-value threshold of 10^−3^) against a database of *Pecten maximus* [66], *Mizuhopecten yessoensis* [89], *Crassostrea gigas* [70] and *SwissProt* proteins obtained from NCBI and UNIPROT:

https://ftp.ncbi.nlm.nih.gov/genomes/refseq/invertebrate/Pecten_maximus/latest_assembly_versions/GCF_902652985.1_xPecMax1.1/GCF_902652985.1_xPecMax1.1_protein.faa.gz (accessed on 28 April 2020)

https://ftp.ncbi.nlm.nih.gov/genomes/archive/old_refseq/Mizuhopecten_yessoensis/protein/protein.fa.gz (accessed on 19 July 2017)

https://ftp.ncbi.nlm.nih.gov/genomes/archive/old_refseq/Crassostrea_gigas/protein/protein.fa.gz (accessed on 06 February 2017)

https://ftp.uniprot.org/pub/databases/uniprot/current_release/knowledgebase/complete/uniprot_sprot.fasta.gz (accessed on 03 October 2020)

Then, annotations were expanded by incorporating information from gene names and functions using gene ontology (GO) and protein structure domains associated with the transcript using InterPro (InterPro. Available online: https://www.ebi.ac.uk/interpro/ (accessed on 15 March 2021)).

Ortholog assignment and pathway mapping were performed on the KEGG Automatic Annotation Server (KAAS, [90]) using Blast and the BBH (bi-directional best hit) method (KAAS—KEGG Automatic Annotation Server. Available online: http://www.genome.jp/tools/kaas/ (accessed on 15 March 2021)).

### 5.8. Protein Network Analysis

To search for the protein-protein interactions, network analyses using the String 10.5 algorithm [91] were performed. The putative human homologs of proteins coded by the up- and down-regulated genes in the *P. maximus* digestive gland were identified by means of a Blastx search [92] against the STRING human protein database (9606.protein.sequences.v10.fa), with an E-value threshold of 10^−5^. The top Blastx search results were used as input in the String program. The up-regulated and the down-regulated genes were analyzed separately.

The genes that code for proteins that showed protein-protein interactions were subjected to GO enrichment analysis with OmicsBox using Fisher’s exact test [93] (up- and down-regulated genes were analyzed separately). The false discovery rate (FDR) adjusted *p*-value [87] was set at a cutoff of 0.05.

### 5.9. Technical Validation of RNA-seq data by RT-qPCR

cDNA was synthesized from 0.5 µg of total RNA with the iScript™cDNA Synthesis kit (ref. 170-8891, BioRad, CA, USA) in a 20 µL reaction volume, and the conditions were 5 min at 25 °C, 30 min at 42 °C and 5 min at 85 °C.

A normalization step using reference genes was performed for the relative expression of gene expression by means of RT-qPCR [94,95,96,97]. Only genes which show stable expression must be employed [39,98].

Four reference gene candidates (Table 7), *GAPDH, EF1A, COX1, NDUFA7*, and six target genes randomly selected (Table 7), *MRP7*, *CYP2B4*, *P5CR*, *SLC6A9*, *FERRITIN*, *CYP2U1*, were used in the gene expression study. The candidate reference genes have been successfully employed previously in *P. maximus* [39]. Oligonucleotide primers (Table 7) were synthesized by Integrated DNA Technologies. The specificity of the primers was confirmed by the presence of a single peak in the melting curve and by the presence of a single band of the expected size when PCR products were run in a 2% agarose gel. The PCR amplification efficiency (E) of each transcript was determined by means of Real Time PCR Miner software (Real-time PCR Miner. Available online: http://www.miner.ewindup.info/ (accessed on 15 March 2021) [99]). The mean amplification efficiency (E) of each amplicon (Table 7) was used in the calculation of gene expression.

Real-time qPCR analysis was conducted in technical duplicates and 6 biological replicates, in 96-well reaction plates on an iCycler iQ^®^ Real-time System (BioRad, CA, USA). The PCR final volume was 20 µL, containing 4 µL of 1:5 diluted cDNA (20 ng of cDNA), 10 µL of SsoFast EvaGreen Supermix (ref. 172-5201, Bio-Rad), 400 nM of forward and reverse primers, and 4.4 µL of PCR-grade water. The cycling conditions were: 30 s at 95 °C (initial template denaturation), and 40 cycles of 5 s at 95 °C (denaturation) followed by 10 s at 60 °C (annealing and elongation) and 10 s at 75 °C for fluorescence measurement. At the end of each run a melting curve was carried out: 95 °C for 20 s and 60 °C for 20 s followed by an increase in temperature from 60 to 100 °C (with temperature increases in steps of 0.5 °C every 10 s). Baseline values were automatically determined for all plates using Bio-Rad iCycler iQ software V3.1 (IQ™ Real-Time PCR Detection System). The threshold value was set manually at 100 RFU to calculate the Cq values. Non-reverse transcriptase controls and non-template controls (NTC) were also included in each run.

Gene expression was normalized to reference genes that had stable expression levels [94,95,96,97]. The gene expression stability of candidate reference genes was analyzed using three Microsoft Excel based software applications, geNorm V3.5 [97], NormFinder V0.953 [94] and BestKeeper V1 [96]. The non-normalized expression (Q) was calculated using the equation Q = (1 + E)^−Cq^. Then the expression was normalized by dividing it by the normalization factor (the geometric mean of the non-normalized expression of the selected reference genes) [39].

### 5.10. Statistical Analyses

The data were log-transformed to meet the requirements of normality and homogeneity of variances. The domoic acid concentration and domoic acid burden in control and treated scallops was compared using Student’s *t*-test. The normalized expression of target genes (log2-transformed) in treated scallops, in relation to the control group, was also compared using Student’s *t*-test. *p* < 0.05 was considered statistically significant. Statistical analyses were carried out with the IBM SPSS Statistics 24.0 package.

## Figures and Tables

**Table 1:** Domoic acid concentration (µg g^−1^ wet weight) and domoic acid burden (µg) in control and treated scallops (*Pecten maximus*). DG, digestive gland; AM, adductor muscle; Other, remaining tissues.

	**Domoic Acid Concentration (µg g−1 Wet Weight): Mean ± SD**					
	**DG**	**Kidney**	**Gonad**	**AM**	**Gill**	**Other**
Control	2222.9 ± 551.7	370.7 ± 238.2	39.0 ± 27.2	35.1 ± 44.9	3.9 ± 3.1	14.4 ± 7.0
Treated	1987.0 ± 406.5	1226.3 ± 1037.3	42.9 ± 17.7	22.9 ± 17.0	6.1 ± 6.9	14.7 ± 8.8
	**Domoic Acid Burden (µg): Mean ± SD**					
	**DG**	**Kidney**	**Gonad**	**AM**	**Gill**	**Other**
Control	3690.2 ± 1002.5	88.8 ± 48.2	51.2 ± 31.4	385.6 ± 526.9	8.7 ± 6.5	114.2 ± 56.6
Treated	3884.8 ± 988.6	329.0 ± 293.3	64.9 ± 30.8	262.4 ± 191.2	13.2 ± 12.7	123.8 ± 62.6

**Table 2:** Summary of the transcriptome assembly for *P. maximus* digestive gland (bp).

Contig N50 Length	1883 bp
Minimum contig length	450 bp
Maximum contig length	18,874 bp
Average contig length	1481 bp
Total length in contigs	107,645,527 bp
Number of assembled unigenes	72,673

**Table 3:** Top 20 up-regulated genes classified by FDR adjusted *p*-value (padj). Only genes with Blastx result are displayed. FC: fold change.

Sequence ID	Description	FC	padj
ci|000200864|proj|Sample_C_D|2	cytochrome P450 2D10-like	16.57	5.88 × 10^−6^
ci|000249823|proj|Sample_C_D|2	probable proline iminopeptidase	14.85	1.33 × 10^−5^
ci|000199464|proj|Sample_C_D|2	pyrroline-5-carboxylate reductase 2-like	2.68	1.57 × 10^−5^
ci|000262059|proj|Sample_C_D|2	integumentary mucin C.1-like	16.32	2.09 × 10^−5^
ci|000180736|proj|Sample_C_D|2	exocyst complex component 4-like	2.67	2.82 × 10^−5^
ci|000220117|proj|Sample_C_D|2	multiple epidermal growth factor-like domains protein 10	14.30	7.17 × 10^−5^
ci|000029875|proj|Sample_C_D|2	cytochrome P450 4F6-like isoform X1	2.48	1.23 × 10^−4^
ci|000033337|proj|Sample_C_D|2	uncharacterized protein LOC117330985	4.40	1.90 × 10^−4^
ci|000031940|proj|Sample_C_D|2	retinol dehydrogenase 7-like isoform X2	2.51	2.59 × 10^−4^
ci|000050118|proj|Sample_C_D|2	4-coumarate--CoA ligase 1-like	2.34	3.48 × 10^−4^
ci|000208120|proj|Sample_C_D|2	46 kDa FK506-binding nuclear protein-like	10.84	3.73 × 10^−4^
ci|000199228|proj|Sample_C_D|2	cholecystokinin receptor-like	11.25	4.78 × 10^−4^
ci|000033677|proj|Sample_C_D|2	ectonucleotide pyrophosphatase/phosphodiesterase family member 5-like isoform X2	8.80	7.96 × 10^−4^
ci|000181387|proj|Sample_C_D|2	sodium- and chloride-dependent glycine transporter 1-like	11.00	8.03 × 10^−4^
ci|000193220|proj|Sample_C_D|2	uncharacterized protein LOC117327200	10.18	1.27 × 10^−3^
ci|000201571|proj|Sample_C_D|2	uncharacterized protein LOC117327200	10.47	1.27 × 10^−3^
ci|000216669|proj|Sample_C_D|2	NPC intracellular cholesterol transporter 2-like	9.15	1.48 × 10^−3^
ci|000211651|proj|Sample_C_D|2	CD109 antigen-like isoform X1	3.45	1.89 × 10^−3^
ci|000199247|proj|Sample_C_D|2	uncharacterized protein LOC117331788	5.65	1.93 × 10^−3^
ci|000027261|proj|Sample_C_D|2	zygotic DNA replication licensing factor mcm3-like	2.31	2.16 × 10^−3^

**Table 4:** Top 20 down-regulated genes classified by FDR adjusted *p*-value (padj). Only genes with blastx result are displayed. FC: fold change.

Sequence ID	Description	FC	padj
ci|000057484|proj|Sample_C_D|2	uncharacterized protein LOC110456411 isoform X5	−56.49	1.06 × 10^−16^
ci|000056978|proj|Sample_C_D|2	uncharacterized protein LOC110456411 isoform X4	−53.9	7.97 × 10^−16^
ci|000094075|proj|Sample_C_D|2	pinin-like	−29.78	2.37 × 10^−11^
ci|000008473|proj|Sample_C_D|2	uncharacterized protein LOC117340902	−3.381	3.65 × 10^−7^
ci|000008352|proj|Sample_C_D|2	uncharacterized protein LOC117325809 isoform X1	−4.911	3.83 × 10^−7^
ci|000077317|proj|Sample_C_D|2	uncharacterized protein LOC117338873	−11.63	4.35 × 10^−7^
ci|000097219|proj|Sample_C_D|2	serine protease inhibitor Cvsi-1-like	−16.21	3.09 × 10^−6^
ci|000161872|proj|Sample_C_D|2	uncharacterized protein LOC117338873	−14.53	2.04 × 10^−5^
ci|000090287|proj|Sample_C_D|2	innexin unc-7-like	−5.491	2.04 × 10^−5^
ci|000055679|proj|Sample_C_D|2	putative nuclease HARBI1	−15.75	2.44 × 10^−5^
ci|000057152|proj|Sample_C_D|2	uncharacterized protein LOC117338914	−11.88	3.84 × 10^−5^
ci|000193936|proj|Sample_C_D|2	uncharacterized protein LOC117332862, partial	−7.373	7.17 × 10^−5^
ci|000071556|proj|Sample_C_D|2	serine/threonine-protein kinase PINK1, mitochondrial-like	−13.21	1.65 × 10^−4^
ci|000038909|proj|Sample_C_D|2	sphingomyelin synthase-related protein 1-like	−4.889	2.27 × 10^−4^
ci|000061087|proj|Sample_C_D|2	uncharacterized protein LOC110461911 isoform X1	−10.47	2.35 × 10^−4^
ci|000058655|proj|Sample_C_D|2	apoptosis-stimulating of p53 protein 1-like	−3.122	3.73 × 10^−4^
ci|000104877|proj|Sample_C_D|2	uncharacterized protein LOC117339040	−9.715	3.90 × 10^−4^
ci|000017126|proj|Sample_C_D|2	uncharacterized protein LOC117326392	−3.294	4.56 × 10^−4^
ci|000019333|proj|Sample_C_D|2	selenoprotein N-like isoform X1	−1.813	5.94 × 10^−4^
ci|000009958|proj|Sample_C_D|2	uncharacterized protein LOC117345233	−2.103	6.09 × 10^−4^

**Table 5:** List of transcripts coding for putative glutamate receptors in the digestive gland of *Pecten maximus*. FC: fold change; padj: FDR adjusted *p*-value.

Sequence ID	Description	Type	FC	padj
ci|000016850|proj|Sample_C_D|2	XP_033740949.1 metabotropic glutamate receptor 1-like [*Pecten maximus*]	Metabotropic	2.938	0.278
ci|000042484|proj|Sample_C_D|2	XP_033740949.1 metabotropic glutamate receptor 1-like [*Pecten maximus*]	Metabotropic	1.309	0.937
ci|000199286|proj|Sample_C_D|2	XP_033740949.1 metabotropic glutamate receptor 1-like [*Pecten maximus*]	Metabotropic	2.566	0.288
ci|000235714|proj|Sample_C_D|2	XP_033740949.1 metabotropic glutamate receptor 1-like [*Pecten maximus*]	Metabotropic	1.575	NA
ci|000005744|proj|Sample_C_D|2	XP_033744387.1 glutamate receptor-like [*Pecten maximus*]	Ionotropic	1.504	0.714
ci|000012480|proj|Sample_C_D|2	XP_033744387.1 glutamate receptor-like [*Pecten maximus*]	Ionotropic	1.567	0.765
ci|000178783|proj|Sample_C_D|2	XP_033744387.1 glutamate receptor-like [*Pecten maximus*]	Ionotropic	3.428	0.123
ci|000194581|proj|Sample_C_D|2	XP_033744387.1 glutamate receptor-like [*Pecten maximus*]	Ionotropic	1.655	0.845
ci|000000691|proj|Sample_C_D|2	XP_033760483.1 glutamate receptor 3-like [*Pecten maximus*]	Ionotropic	1.242	0.945
ci|000077591|proj|Sample_C_D|2	XP_033760483.1 glutamate receptor 3-like [*Pecten maximus*]	Ionotropic	−1.006	0.998
ci|000108874|proj|Sample_C_D|2	XP_033760483.1 glutamate receptor 3-like [*Pecten maximus*]	Ionotropic	−1.199	0.968
ci|000219160|proj|Sample_C_D|2	XP_033760483.1 glutamate receptor 3-like [*Pecten maximus*]	Ionotropic	−1.319	0.937
ci|000054856|proj|Sample_C_D|2	XP_033760586.1 glutamate receptor U1-like [*Pecten maximus*]	Ionotropic	−1.100	0.970
ci|000106439|proj|Sample_C_D|2	XP_033760586.1 glutamate receptor U1-like [*Pecten maximus*]	Ionotropic	−1.447	0.912
ci|000013492|proj|Sample_C_D|2	XP_033763623.1 metabotropic glutamate receptor 7-like isoform X3 [*Pecten maximus*]	Metabotropic	1.042	0.985
ci|000015386|proj|Sample_C_D|2	XP_033763623.1 metabotropic glutamate receptor 7-like isoform X3 [*Pecten maximus*]	Metabotropic	1.066	NA
ci|000028888|proj|Sample_C_D|2	XP_033763623.1 metabotropic glutamate receptor 7-like isoform X3 [*Pecten maximus*]	Metabotropic	1.169	0.937
ci|000048793|proj|Sample_C_D|2	XP_033763623.1 metabotropic glutamate receptor 7-like isoform X3 [*Pecten maximus*]	Metabotropic	1.161	0.928
ci|000191381|proj|Sample_C_D|2	XP_033763623.1 metabotropic glutamate receptor 7-like isoform X3 [*Pecten maximus*]	Metabotropic	1.348	0.937

**Table 6:** Summary of the functional annotation results.

Functional Annotation	Number	%
**Differentially expressed unigenes**	**535**	**100**
With Blastx hit	286	53.5
With GO terms	259	48.4
With enzyme code	129	24.1
With KO ortholog	102	19.1
With PFAM domains	188	35.1
**All unigenes**	**72,673**	**100**
With Blastx hit	35,583	49.0
With GO terms	32,203	44.3
With enzyme code	16,429	22.6
With KO ortholog	7657	10.5
With PFAM domains	24,810	34.1

**Table 7:** Genes selected for RT-qPCR: sequence names, description, gene symbols, primers, amplicon length (bp) for each primer pair and average efficiency (E).

Sequence Name	Description	Symbol	Sense Primer	Antisense Primer	bp	E
ci|000200326|proj|Sample_C_D|2	glyceraldehyde-3-phosphate dehydrogenase	*GAPDH*	TCCGGATGTGTCTGTTGTTGAC	TTCAGATCTCCATCAGCTGCAC	102	0.8465
ci|000017232|proj|Sample_C_D|2	eukaryotic translation elongation factor 1 alpha	*EF1A*	AGGGCTCCTTCAAGTATGCCTG	TGAGCGGTCTCGAACTTCCAC	100	0.8275
ci|000019926|proj|Sample_C_D|2	cytochrome c oxidase subunit 1	*COX1*	AGTGGAGAACTATTGGGTGTGC	AGACCTAGGCCGATTTCCAAAC	119	0.8525
ci|000005190|proj|Sample_C_D|2	NADH dehydrogenase (ubiquinone) 1 alpha subcomplex subunit 7	*NDUFA7*	ATTACACACGAGATGGACGCTG	ACATCAGAGCTGGCTGTTTCAG	115	0.8043
ci|000002411|proj|Sample_C_D|2	multidrug resistance-associated protein 7	*MRP7*	CGGATGGTGGCTAACTCATT	CGATGCACCCATACACTGTC	200	0.8594
ci|000062123|proj|Sample_C_D|2	cytochrome p450 2b4-like	*CYP2B4*	CATGCGAAGGACTACGACAAG	GAACAAAATGGCCCAAAGAAG	183	0.8314
ci|000199464|proj|Sample_C_D|2	pyrroline-5-carboxylate reductase 2-like	*P5CR*	CCTCACATCATCACTCCA GTCC	GACGGGAGCAGATTCTCCTC	119	0.8547
ci|000181387|proj|Sample_C_D|2	sodium- and chloride-dependent glycine transporter 1-like	*SLC6A9*	GACGGTACTGGGCATTTCTG	ATCAGCAAGGCCGTAAGGAG	183	0.8539
ci|000018690|proj|Sample_C_D|2	ferritin 2	*FERRITIN*	CCATGCTGAAACCGAGGCTG	CAATCCTGCCTCCTCTCTTG	206	0.8547
ci|000200864|proj|Sample_C_D|2	cytochrome p450 2u1-like	*CYP2U1*	CATCGACGCCTTCCAGTTCG	GATAGTCAGCCATCGTGGGT	233	0.8753

**Table 8:** Rank of four candidate reference genes in quantitative real-time reverse transcription–polymerase chain reaction (RT–qPCR), calculated by geNorm, NormFinder, and BestKeeper analysis.

Rank	geNorm (Average M)		NormFinder (Stability)		BestKeeper (*r*)		BestKeeper (SD)	
1	*GAPDH-COX1*	0.78	*GAPDH*	0.105	*COX1*	0.858	*GAPDH*	0.724
2	*GAPDH-COX1*	0.78	*COX1*	0.196	*GAPDH*	0.782	*NDUFA7*	0.781
3	*EF1A*	1.01	*EF1A*	0.244	*EF1A*	0.662	*COX1*	0.838
4	*NDUFA7*	1.34	*NDUFA7*	0.386	*NDUFA7*	−0.243	*EF1A*	0.861

## Data Availability

The data presented in this study are openly available in the NCBI Sequence Read Archive (BioProject ID PRJNA704533, BioSample accessions: SAMN18043529 to SAMN18043540) and in the Supplementary Materials.

## Supplementary Materials

The following are available online at https://www.mdpi.com/article/10.3390/toxins13050339/s1, Table S1: Summary of BUSCO analysis results obtained in the transcriptome of *Pecten maximus* digestive gland using the Metazoa database (metazoa_odb10), Figure S1: MA plot showing log2 fold-change as a function of mean log expression level. The red dots represent genes with adjusted *p*-value < 0.05 and FC > 1.5 or <−1.5 (DEGs); the grey dots represent non-DEGs, Figure S2. Volcano plot. The red dots represent genes with adjusted *p*-value < 0.05 and FC > 1.5 or <−1.5 (DEGs); the grey dots represent non-DEGs, Figure S3: Species distribution of the top Blastx hits, File S1: List of differentially expressed genes. Sequence name, description, fold change (FC), FDR adjusted *p*-value (padj) and annotation results are shown, File S2: Nucleotide sequences of differentially expressed genes (in fasta format), File S3: List of transcripts coding for putative glutamate receptors in the digestive gland of *Pecten maximus*, File S4: Annotation of the transcripts expressed in the digestive gland of *P. maximus*. File S5: List of KO (KEGG Orthologs) for the differentially expressed genes and for all genes, File S6: Results of a Blastx search of up-regulated genes against the STRING human protein database (9606.protein.sequences.v10.fa), and list of input proteins in STRING network analysis, File S7: Results of a Blastx search of down-regulated genes against the STRING human protein database (9606.protein.sequences.v10.fa), and list of input proteins in STRING network analysis, File S8: Significantly enriched GO terms in the genes that code for proteins that showed interactions in the protein network analysis. The first two spreadsheets list the enriched GO terms for the up-regulated and the down-regulated genes respectively, the next two spreadsheets show the enriched GO terms after GO slim.
