# Supplementary material for: Transcriptional Response in the Digestive Gland of the King Scallop (Pecten maximus) After the Injection of Domoic Acid
Source: Toxins (Basel). 2021 May 7;13(5):339. doi: 10.3390/toxins13050339 (PMC8150855; doi:10.3390/toxins13050339)
Supplement: Supplementary file 1 [file toxins-13-00339-s001.zip › toxins-supplementary table and figure.pdf]

# Supplementary Materials: Transcriptional Response in the Digestive Gland of the King Scallop (*Pecten maximus*) After the Injection of Domoic Acid

**Pablo Ventoso, Antonio J. Pazos, Juan Blanco, M. Luz Pérez-Parallé, Juan C. Triviño and José L. Sánchez**

**Table S1.** Summary of BUSCO analysis results obtained in the transcriptome of *Pecten maximus* digestive gland using the Metazoa database (metazoa_odb10).

| BUSCO Notation | Percentage of BUSCOs | Number of BUSCOs |
|---|---|---|
| Complete Single-copy | 45.60 | 435 |
| Complete Duplicated | 36.69 | 350 |
| Fragmented | 3.88 | 37 |
| Missing | 13.84 | 132 |

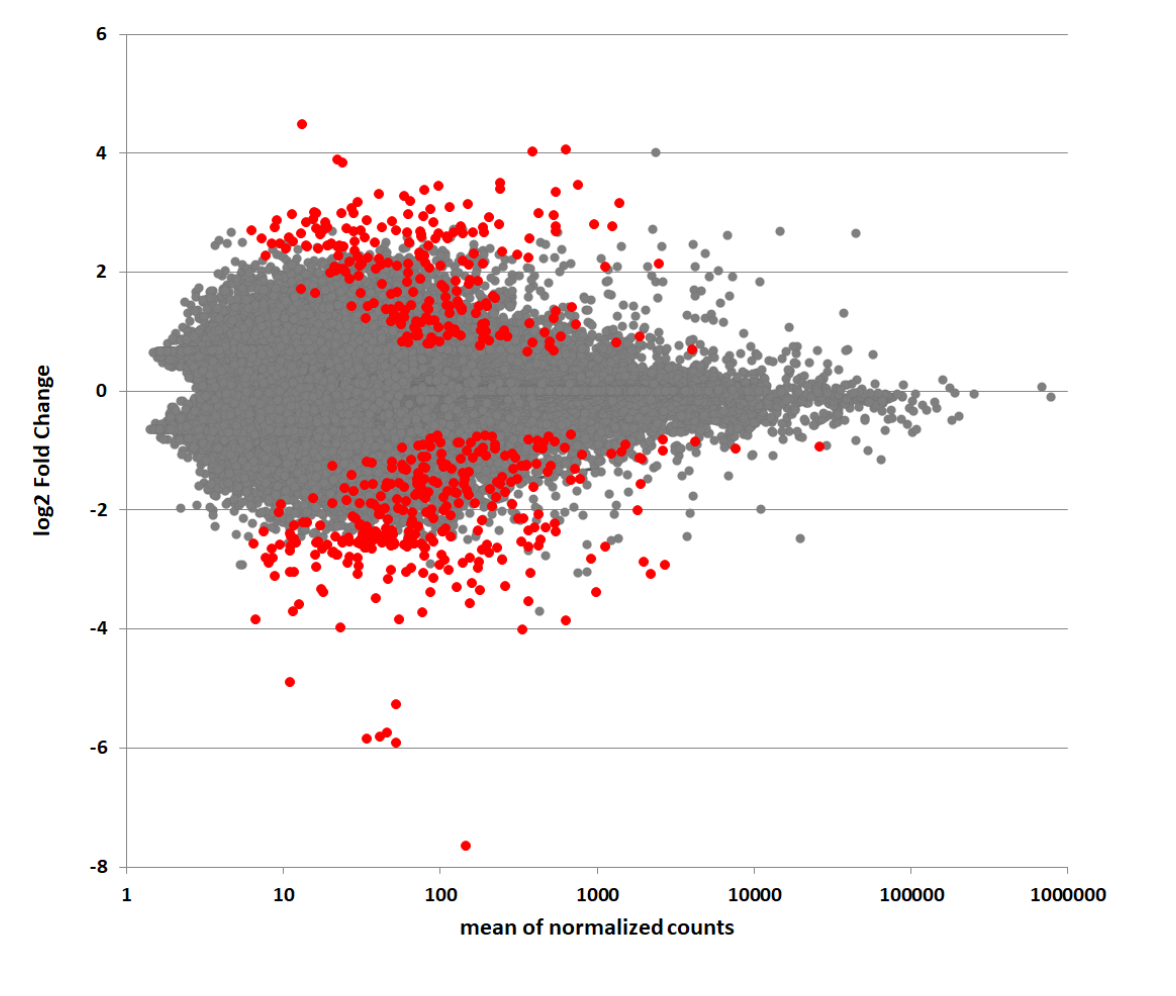


**Figure S1.** MA plot showing log2 fold-change as a function of mean log expression level. The red dots represent genes with adjusted *p*-value <0.05 and FC > 1.5 or < −1.5 (DEGs); the grey dots represent non-DEGs.

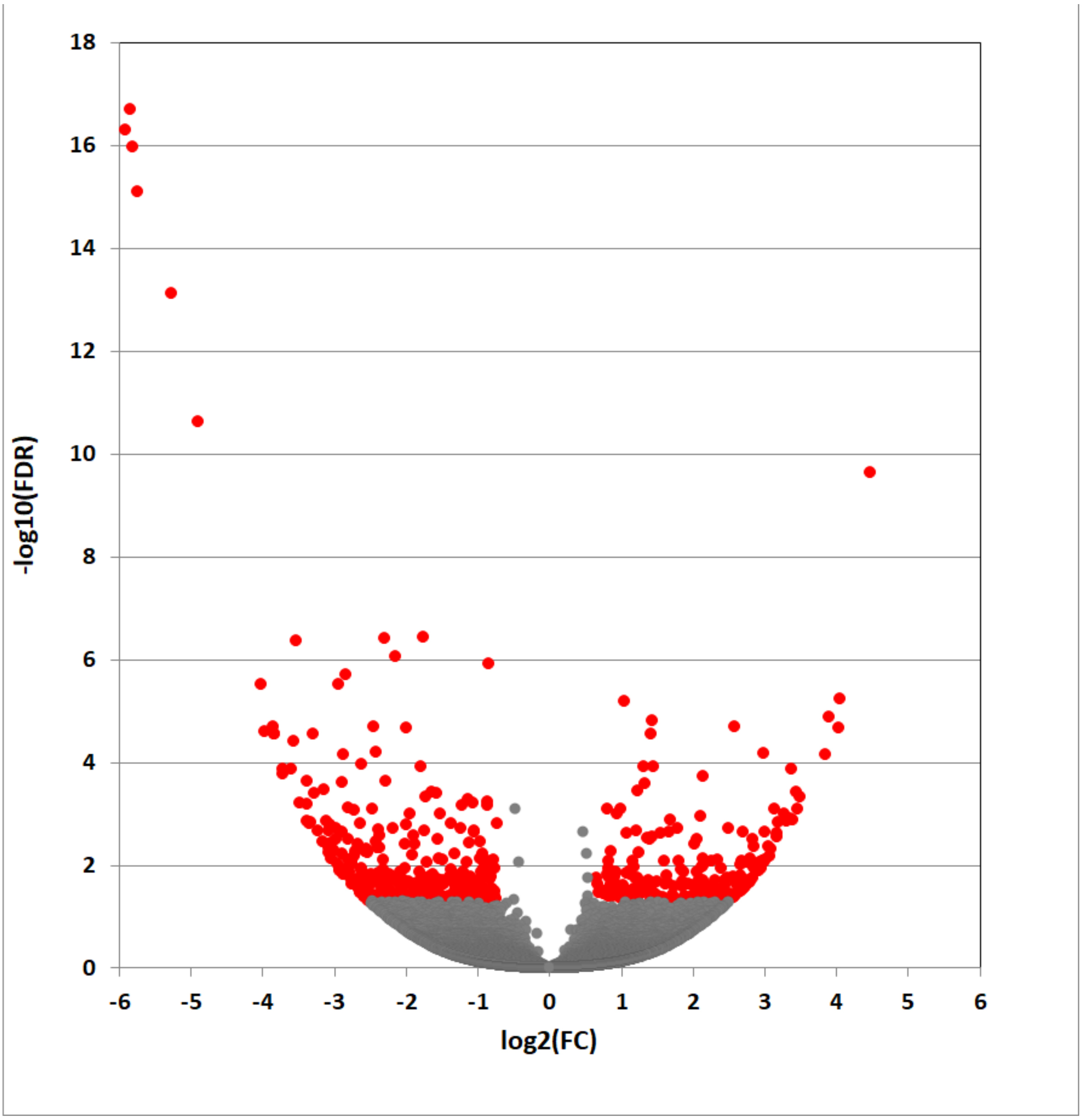


**Figure S2.** Volcano plot. The red dots represent genes with adjusted *p*-value <0.05 and FC > 1.5 or < −1.5 (DEGs); the grey dots represent non-DEGs.

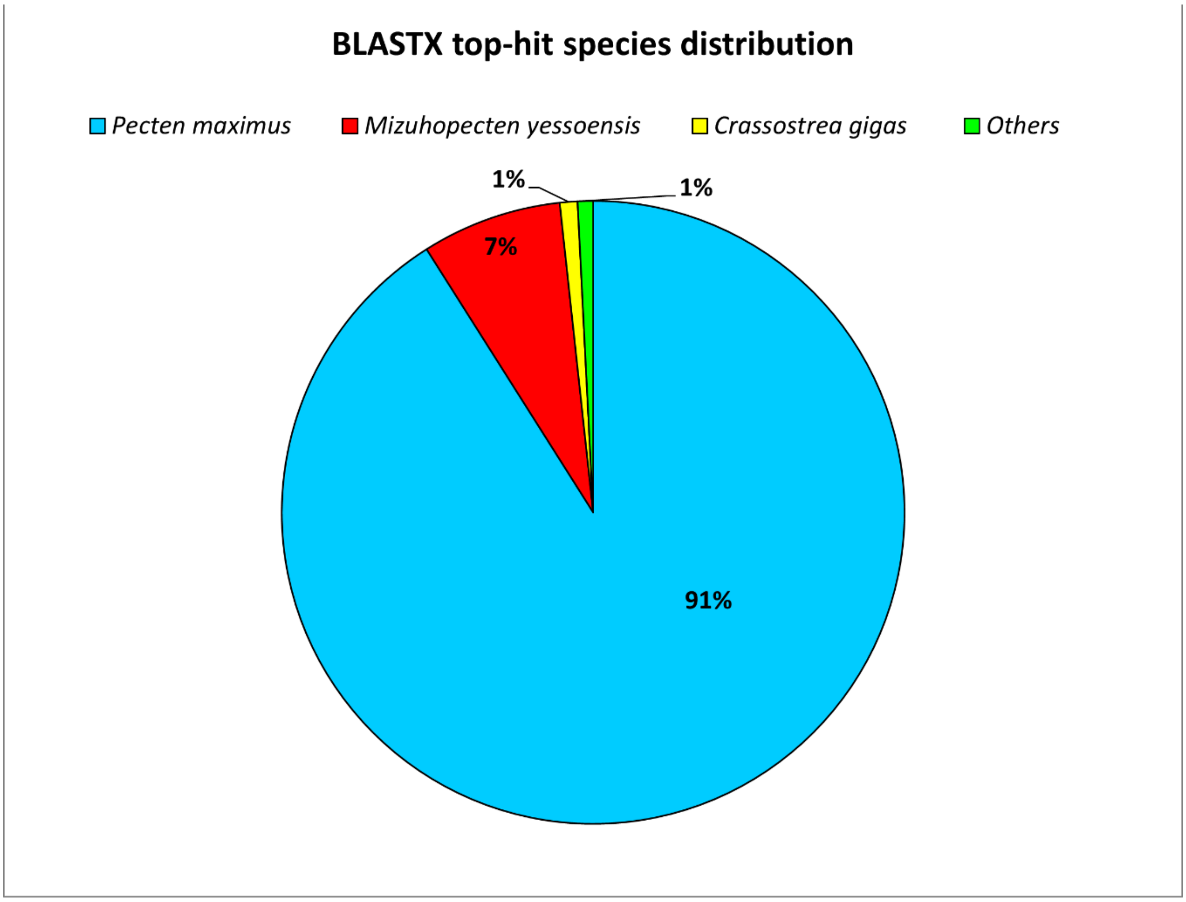


**Figure S3.** Species distribution of the top Blastx hits.
